# Use of Artificial Intelligence in obtaining canine diets: nutritional inadequacy and the need for technical knowledge

**DOI:** 10.1016/j.vas.2026.100761

**Published:** 2026-07-20

**Authors:** M.B.S. Rosa, J. França, M.T.S. Siqueira, C. Raineri

**Affiliations:** a School of Veterinary Medicine and Animal Science, Federal University of Uberlândia, Uberlândia, MG, 38410-337, Brazil; b Department of Animal Science, São Paulo State University, Jaboticabal, SP, 14884-900, Brazil; c Faculty of Animal Science and Food Engineering, São Paulo University, Pirassununga, SP, 13630-000, Brazil

**Keywords:** Canine nutrition, Chat GPT, diet formulation, Gemini, Nutritional deficiencies

## Abstract

This study evaluated the nutritional adequacy of dog diets formulated by four widely used Artificial Intelligence models (Manus, ChatGPT, DeepSeek, and Gemini) against the recommendations of the European Pet Food Industry Federation. This was a theoretical, in silico study, no animals were fed or clinically evaluated. Using a standardized prompt, complete and balanced diets (beef and chicken) for an 8 kg adult dog were requested. Eleven formulations were analyzed using SuperCracPet® software based on United States Department of Agriculture data. Overall, 61.3% of nutrients were below the minimum recommended levels. Nutrient levels below FEDIAF minimum requirements were identified across multiple categories: energy supply was insufficient in 81% of diets, calcium levels were below recommendations in 91% of formulations, calcium-to-phosphorus ratios were outside the recommended range in 63% of diets, chlorine was absent in all formulations, vitamin D levels were below requirements in 63.7% of diets, choline in 81.8%, and linoleic acid in 72%. Only 18% of the formulations included supplementation, which was provided in insufficient quantity. If implemented chronically, these nutritional inadequacies may pose risks to bone, dermatological, immune, and metabolic health. It is concluded that, in their current form, the evaluated language models are insufficient for the autonomous formulation of canine diets, as the generated prescriptions presented multiple nutritional inadequacies. Safe application requires hybrid systems with mandatory supervision by animal scientists and veterinarians, as well as regulation to prevent avoidable risks to animal health.

## 1 Introduction

Nutrition is a fundamental pillar in maintaining the health, longevity, and quality of life of dogs, being considered the "fifth vital sign" in veterinary clinical evaluation (Freeman et al., 2011). A complete and balanced diet plays a crucial role in disease prevention, immune system support, and optimization of physiological performance (Rosa, 2022). Dietary formulation should be based on solid scientific evidence and consider the specific nutritional needs of the species, life stage, and individual health condition of the animal (Rosa, 2022).The risks associated with inadequate formulations are significant, ranging from nutrient deficiencies and excesses to critical imbalances, such as the calcium-phosphorus ratio, which can lead to serious health problems (Pedrinelli et al., 2017).

Historically, dog feeding has transitioned from empirical homemade diets to the widespread adoption of commercial dog food, culminating in a growing interest in balanced natural diets (Tazerji et al., 2024). Modern diet formulation is a technical and precise process that requires the use of nutritional composition tables and strict adherence to recommendations established by international regulatory bodies such as the National Research Council (NRC), the European Pet Food Industry Federation (FEDIAF), and the Association of American Feed Control Officials (AAFCO) (FEDIAF, 2024; NRC, 2006). Technical precision is vital. Furthermore, analysis of published homemade recipes has shown that most have deficiencies in multiple essential nutrients, such as iron, vitamin E, zinc, and calcium, expose animals to nutritional risks (Pedrinelli et al., 2017).

Technological advancements have driven the use of digital tools and Artificial Intelligence (AI) in various health sectors, including human and veterinary medicine (Zhang et al., 2023; Zheng et al., 2024). AI, which encompasses machine learning (ML) and natural language processing, has the potential to process large volumes of data (genomic, phenotypic, and behavioral) to generate insights and support professional decision-making (Mwaura, 2024; Sharma and Gaur, 2024). In the area of animal health, AI already demonstrates potential in several applications, such as diagnosis, welfare monitoring, and optimization of feed formulations (Akinsulie et al., 2024; Kumar and Sharma, 2025).

Chatbots based on Large Language Models (LLM), such as ChatGPT and Gemini, are AI systems capable of simulating human conversations and generating technical texts quickly and in context (Papastratis et al., 2024). These tools are trained on vast text datasets, giving them the ability to produce recommendations and plans in various areas (Kassem et al., 2025). In human nutrition, chatbots are already used for dietary assessment, personalized meal planning, and providing nutritional recommendations (Papastratis et al., 2024). Although they demonstrate potential for generating diet plans with high overall quality and caloric accuracy, studies in humans already point to limitations, especially in the balance of macronutrients and fatty acids (Kaçar et al., 2025; Papastratis et al., 2024).

Interest in using AI to formulate diets for companion animals has grown exponentially among pet owners and professionals (OVC Pet Nutrition, 2025); however, the current scenario is marked by a scarcity of scientific studies evaluating the safety and efficacy of the direct use of chatbots in formulating diets for dogs and cats. The application of ML in the pet food industry focuses mainly on ingredient optimization and quality control and not on generating complete recipes for the end consumer (Kumar and Sharma, 2025). The lack of technical validation for AI-generated diets represents a risk, as the linguistic precision of the models can mask nutritional imprecision, exposing animals to incomplete diets.

The formulation of diets by chatbots, especially in clinical and nutritional contexts, presents potential flaws that compromise safety. Studies demonstrate that LLM models can generate diets with notable deficiencies in specific nutrients and fail to provide adequate personalization (Onay et al., 2025). An additional and inherent risk of these models is the occurrence of AI "hallucinations," where the chatbot generates incorrect or fabricated information, which can be dangerous when applied to health recommendations (Shiferaw et al., 2024). Such failures include nutritional calculation errors, disregard for nutrient bioavailability, and inadequate generalizations that ignore the clinical individuality of the patient (Ponzo et al., 2024). The moderation in chatbot accuracy, observed in human nutrition studies, is even more critical in veterinary nutrition, where the margins of error for strict carnivores and dogs are narrow.

The rapid adoption of AI tools in diet formulation, coupled with a lack of validation studies, creates an urgent scientific gap. There are no robust comparative studies that assess and contrast nutritional quality, adequacy to current recommendations (NRC, FEDIAF), and safety of diets formulated by different AI chatbots against established animal nutrition standards. Identifying and quantifying nutritional deficits and excesses in these diets is crucial to inform the scientific community, veterinary and animal science professionals, and pet owners about the real risks of AI-assisted self-prescription diets. Given this scenario, the present study aimed to evaluate and compare dog diets formulated by different Artificial Intelligence chatbots (Manus, ChatGPT, DeepSeek, and Gemini) to analyze their nutritional adequacy against current recommendations, focusing on energy, protein, vitamin, and mineral intake windows.

## 2 Materials and methods

### 2.1 Experimental design and AI models evaluated

This analytical study was conducted in December 2025 with the objective of evaluating the nutritional adequacy of dog diets formulated by large-scale language models. Four widely used AI systems were evaluated and accessed in their publicly available free versions: Manus (Butterfly Effect), ChatGPT-40 (OpenAI), Gemini 2.0 Flash Experimental (Google), and DeepSeek-V3 (DeepSeek AI). All prompts were entered in Brazilian Portuguese.

### 2.2 Standardized prompt protocol

For methodological standardization and elimination of formulation bias, all AI models received exactly the same textual prompt, without additional interactions, adjustments, or subsequent refinements. The prompt used was:

"*Create two complete and balanced diets (one based on beef and the other based on chicken) of natural food for a healthy adult dog weighing 8kg that has little stimulation for physical activity. Provide me with the quantity of each ingredient in the diet to be given daily and remember: It is a complete and balanced diet*."

This single-interaction approach was intentionally chosen to simulate typical use by lay owners, who may request complete formulations without additional technical information. In Brazil, there is a preference for small dogs, reflecting urban spatial limitations and the search for animals adaptable to apartments (Bradley et al., 2022; PetConect, 2025; Rodrigues et al., 2023). Small dogs are defined as those weighing between 5 kg and 10 kg (Salt et al., 2017), therefore the hypothetical weight of 8 kg was established to represent this reality.

Each model was asked to formulate two distinct diets (beef and chicken based). When the same model spontaneously generated multiple versions for the same protein source, offering alternative ingredient options, all formulations were retained for intra-model variability analysis, totaling 11 diets analyzed.

### 2.3 Analysis of the formulated diets

The diets were analyzed exactly as provided by the AI models without corrections, technical adjustments, exclusion of ingredients, or addition of supplements, respecting the principle of evaluating the real performance of these tools when used directly by non-specialized users. Each complete formulation (list of ingredients and daily quantities) was entered into the SuperCracPet® software version 1.3 (TD Software, Viçosa, MG, Brazil), specialized in the formulation and nutritional evaluation of diets for dogs and cats (TD Software, 2020). The software uses a food composition database from the United States Department of Agriculture (USDA) and was used to provide the complete nutrient profile of the diets and detailed estimates of metabolizable energy (ME).

### 2.4 Calculation of energy requirements

The daily energy requirement of the hypothetical dog (adult, healthy, 8 kg, low level of physical activity) was calculated using the validated metabolic equation for adult dogs in maintenance (NRC, 2006):

(1) $$\text{DER}\left(\text{kcal}/\text{day}\right)=95\times BW^{0,75}$$

Where DER = daily energy requirement and BW = body weight in kg.

For the 8 kg animal, the calculated energy requirement was 451.9 kcal/day. This value was used as a reference to assess the energy supply of each formulated diet, which allowed the identification of caloric deficit or surplus.

### 2.5 Nutritional reference standard

The nutritional results obtained in SuperCracPet® were compared to the nutritional recommendations for adult dogs in maintenance established by the FEDIAF (2024), which provides standardized reference values expressed per 1000 kcal of ME (Table 1). FEDIAF was chosen as the standard because it represents a widely validated and widely used international scientific consensus in the pet food industry and in veterinary clinical practice.

**Table 1:** Nutritional Recommendations for Adult Dogs in Maintenance^1^.

Nutrient	Unit	Min	Max	Nutrient	Unit	Min	Max
PROTEIN	G	52.10	-	VITAMINS			
Arginine	G	1.51	-	Vitamin A	UI	1754.00	100,00.00
Histidina	G	0.67	-	Vitamin D	UI	159.00	800.00
Isoleucine	G	1.33	-	Vitamin E	UI	10.40	-
Leucine	G	2.37	-	Vitamin B1	mg	0.62	-
Lysine	G	1.22	-	Vitamin B2 (Riboflavin)	mg	1.74	-
Methionine	G	1.16	-	Vitamin B3 (Niacin)	mg	4.74	-
Methionine+cystine	G	2.21	-	Vitamin B5 (Pantothenic acid)	mg	4.11	-
Phenylalanine	G	1.56	-	Vitamin B6 (Pyridoxine)	mg	0.42	-
Phenylalanine+tyrosine	G	2.58	-	Vitamin B8 (Choline)	mg	474.00	-
Threonine	G	1.51	-	Vitamin B9 (Folic acid)	mg	0.08	-
Tryptophan	G	0.49	-	Vitamin B12	mg	0.01	-
Valine	G	1.71	-				
				MICROELEMENTS			
MINERALS				Copper	mg	2.08	-
Calcium	G	1.45	6.25	Iodine	mg	0.30	-
Phosphorus	G	1.16	4.00	Iron	mg	10.40	-
Ca:P ratio		1:1	1:2	Manganese	mg	1.67	-
Potassium	G	1.45	-	Selenium	mg	0.09	-
Sodium	G	1.45	-	Zinc	mg	20.80	-
Chlorine	G	0.43	-				
Magnesium	G	0.20	-	FAT	g	13.75	-
				Linoleic acid	g	3.82	-

Min: minimum; max: maximum.

1 Values expressed per 1000 kcal of metabolizable energy Source: FEDIAF (2024).

### 2.6 Adjustment of recommendations by energy density

Since the diets analyzed presented variable energy densities (900–1769 kcal/kg), the FEDIAF recommendations were adjusted proportionally to the specific metabolizable energy of each diet, using a simple rule of three:

(2) Adjustedrequirement=(FEDIAFrequirement/1000)×dietaryME

Where ME = metabolizable energy in kcal provided by the specific diet.

This adjustment allowed for a precise assessment of the percentage of fulfillment, deficit, or excess of each nutrient in relation to official recommendations, considering that animals consume food to meet energy needs, not a fixed volume of food.

### 2.7 Descriptive statistical analysis

The data were organized in a descriptive and comparative manner, considering the evaluation of the nutritional components provided by the formulated diets. The levels of crude protein, ether extract, fatty acid profile, amino acids, vitamins, and minerals were analyzed in light of the nutritional requirements recommended by FEDIAF (2024) (Table 1), using the rule of three to calculate the percentage of fulfillment and the respective nutritional deficit or excess. The percentages of energy and nutritional fulfillment were calculated as:

(3) %Attendance=(Providedvalue/Adjustedrecommendedvalue)×100

Values <100% indicate a deficit (under-service), values >100% indicate excess (over-service), and values between 100–110% were considered adequate, respecting a nutritional safety margin of 10%, for all evaluated nutrients and dietary items. All calculations were performed using Microsoft Excel® software (Microsoft Corporation, 2021).

### 2.8 Ethical considerations

This study used publicly available, internet-sourced data and was based exclusively on an silico analysis of dietary formulations generated by AI systems. It did not involve either human or animal participants and therefore did not require institutional ethics committee approval. No personal information was used, and the study was conducted in accordance with the Brazilian general law for the protection of personal data (Law No. 13.709/2018).

## 3 Results

### 3.1 General characterization of diets formulated by AI models

The four AI models generated 11 diets with distinct compositions, five based on beef (Table 1) and six based on chicken (Table 2), which showed variations in ingredient selection, total prescribed amounts, and the inclusion of supplementation.

**Table 2:** Ingredient inclusion (g/day) of beef-based diets formulated by AI models.

Ingredient	Manus	ChatGPT1	ChatGPT2	DeepSeek	Gemini
Boiled beef shank	140.00	120.00	120.00	-	-
Raw beef shank	-	-	-	200.00	-
Cooked beef muscle	-	-	-	-	125.00
Cooked Chicken neck	20.00	-	-	-	-
Cooked beef liver	10.00	-	-	-	15.00
Raw beef liver	-	-	-	25.00	-
Boiled beef heart	10.00	-	-	-	-
Raw beef heart	-	-	-	25.00	-
Cooked brown rice	-	45.00	45.00	-	-
Cooked carrots	6.67	20.00	20.00	60.00	16.67
Cooked zucchini	6.67	-	-	-	16.67
Cooked pumpkin	-	40.00	40.00	125.00	-
Cooked broccoli	6.67	-	-	65.00	16.67
Fish oil	2.50	-	5.00		
Olive oil	-	5.00	-	2.50	5.00
Brewer’s yeast	5.00	-	-	-	85.00
Eggshell	-	1.20	1.20	-	-
Vetnil® supplement	-	0.80	0.80	-	-
Total (g)	207.51	237.00	237.00	502.50	280.01

ChatGPT offered the option of using two different fat sources (fish oil and olive oil) in the same formulation, resulting in the analysis of two independent diets.

To ensure a comprehensive analysis of the variability of the AI ​​models, some diets were broken down into multiple formulations according to the ingredient options suggested by the chatbots themselves. In the case of the beef diet, ChatGPT offered the option of using two different fat sources (fish oil and olive oil) in the same formulation, resulting in the analysis of two independent diets (Table 3).

**Table 3:** Ingredient inclusion (g/day) of chicken-based diets formulated by AI models.

Ingredient	Manus1	Manus2	ChatGPT1	ChatGPT2	DeepSeek	Gemini
Cooked Chicken breast	140.00	-	130.00	130.00	-	130.00
Cooked Chicken thigh	-	140.00	-	-	-	-
Raw Chicken thigh/drumstick with ground bone		-	-	-	175.00	-
Cooked Chicken neck	20.00	20.00	-	-	-	-
Cooked beef liver	10.00	10.00	-	-	-	-
Raw Chicken liver	-	-	-	-	25.00	-
Boiled beef heart	10.00	10.00	-	-	-	-
Raw Chicken heart	-	-	-	-	50.00	-
Coocked Chicken gizzards	-	-	-	-	-	15.00
Boiled Sweet potatoes	-	-	50.00	50.00	125.00	-
Boiled yam	-	-	-	-	-	75.00
Coooked carrots	6.67	6.67	-	-	-	-
Cooked zucchini	6.67	6.67	20.00	20.00	-	-
Cooked spinach	-	-	-	-	60.00	-
Boiled chayote	-	-	-	-	65.00	20.00
Cooked broccoli	6.67	6.67	30.00	30.00	-	-
Cooked cabbage	-	-	-	-	-	20.00
Cooked green beans	-	-	-	-	-	20.00
Fish oil	2.50	2.50	5.00	-	-	-
Coconut oil	-	-	-	-	-	5.00
Olive oil	-	-	-	-	2.50	-
Flaxseed oil	-	-	-	5.00	-	-
Brewer’s yeast	5.00	5.00	-	-	-	-
Eggshell	-	-	1.20	1.20	-	-
Vetnil® supplement	-	-	0.80	0.80	-	-
Total (g)	207.51	207.51	237.00	237.00	502.50	285.00

Manus model suggested using two protein sources (chicken breast or thigh), while ChatGPT offered two fat options (fish oil or flaxseed oil).

Similarly, for the chicken diet the Manus model suggested using two protein sources (chicken breast or thigh), while ChatGPT offered two fat options (fish oil or flaxseed oil). These variations were analyzed separately to assess the impact of the changes on final nutritional adequacy.

The DeepSeek model prescribed a substantially higher daily volume than the others and was the only one to suggest raw ingredients. Only the ChatGPT model included supplementation (1.2 g eggshell and 0.8 g Vetnil® supplement), while the Manus, DeepSeek, and Gemini models did not include any form of vitamin-mineral supplementation.

### 3.2 Nutritional compliance and variability

The analysis of the nutritional composition of these diets revealed substantial variability among models and between protein sources (Table 4). All models met the minimum recommended intake of crude protein and fat. Mean crude protein across beef-based diets was 112.6 ± 11.8 g/1000 kcal, and across chicken-based diets was 130.6 ± 16.4 g/1000 kcal — both well above the FEDIAF (2024) minimum of 52.1 g/1000 kcal. Mean ether extract was 42.8 ± 1.4 g/1000 kcal for beef-based and 34.9 ± 7.0 g/1000 kcal for chicken-based diets, both exceeding the minimum requirement of 13.8 g/1000 kcal.

**Table 4:** Nutritional requirements and levels of adequacy for the hypothetical dog in the diets generated by AI chatbots, for beef- and chicken-based formulations^1^.

	Requirement	Manus1	Manus2	ChatGPT1	ChatGPT2	DeepSeek	Gemini	Mean	SD
*Beef*									
Crude protein	52.10	135.80	-	100.2	104.30	119.00	103.90	112.60	11.80
EE	13.80	42.90	-	45.00	44.10	41.00	41.10	42.80	1.40
Ca:P Ratio^2^	1:1 – 2:1	0.04	-	1.10	1.10	2.70	0.10	1.00	0.70
ME	-	1661.70	-	1460.00	1403.00	900.00	1501.00	-	-
*Chiken*									
Crude protein	52.10	168.20	121.50	133.60	139.40	99.30	121.90	130.60	16.40
EE	13.80	26.90	49.70	31.60	33.00	41.20	27.00	34.90	7.00
Ca:P Ratio	2:1	0.08	0.09	1.20	1.20	0.30	0.20	0.50	0.50
ME	-	1506.00	1769.00	1326.00	1271.00	1004.00	1354.00	-	-

ME: metabolizable energy EE: ethereal extract; SD: standard deviations;.

1 values in g/1000 kcal, except ME in kcal/kg;.

2 Ca:P values represent the recommended range (minimum 1:1, maximum 2:1) per FEDIAF (2024).

### 3.3 Meeting energy needs

Energy adequacy in relation to the calculated requirement (451.9 kcal/day) indicated that nine of the eleven diets presented an energy undersupply, with deficits ranging from −31.6 to −150.7 kcal/day. Only the DeepSeek model met the energy requirement in the beef-based diet and resulted in energy oversupply in the chicken-based diet, whereas the ChatGPT and Manus models prescribed a fixed feed volume regardless of protein source or resulting energy density (Table 5). The energy requirement of 451.9 kcal/day was calculated using the NRC (2006) equation for an 8 kg sedentary adult dog and represents a population mean, individual variation means that actual requirements for specific animals may differ. The AI-generated diets ranged from 301.2 to 504.5 kcal/day (Table 5), with 9 of 11 diets falling below the reference value, representing potential deficits of up to 33.4% (150.7 kcal/day).

**Table 5:** Energy contents and levels of adequacy for the hypothetical dog in the diets generated by AI chatbots, for beef- and chicken-based formulations^1^.

Model	Total volume (g/day)	Metabolic energy provided (kcal/day)	Attendance (%)	Deficit/Excess (kcal/day)
*Beef*				
Manus	207.50	344.70	76.30%	−107.20
ChatGPT1	237.00	346.00	76.60%	−105.90
ChatGPT2	237.00	332.50	73.60%	−119.40
DeepSeek	502.50	452.30	100.10%	+0.50
Gemini	280.00	420.30	93.00%	−31.60
*Chicken*				
Manus1	207.50	312.50	69.10%	−139.40
Manus2	207.50	367.00	81.20%	−89.40
ChatGPT1	237.00	314.30	69.50%	−137.60
ChatGPT2	237.00	301.20	66.70%	−150.70
DeepSeek	502.50	504.50	111.60%	+52.60
Gemini	285.00	386.00	85.40%	−65.90

1 considering the recommendation of 451.9 kcal/day (FEDIAF, 2024).

### 3.4 Essential fatty acid profile

Analysis of the formulations generated by ChatGPT (Table 6), revealed that the choice of lipid source, although seemingly a detail, resulted in significant variations in ME and, crucially, in the omega-6:omega-3 ratio. Eight of the 11 diets (72%) showed a linoleic acid deficiency. The n-6:n-3 ratios were inadequate in 63% of the diets and high values ​​in 5 diets, characterizing a pro-inflammatory profile (Table 7).

**Table 6:** Impact of the lipid source on the formulation by ChatGpt.

Diet	Lipid source	Metabolizable energy (kcal)	n-6:n-3 ratio
Beef 1	Olive oil	1460.00	4.87:1
Beef 2	Fish oil	1403.00	3.27:1
Chicken 1	Linseed oil	1326.00	0.56:1
Chicken 2	Fish oil	1271.00	3.49:1

**Table 7:** Essential fatty acid contents and levels of adequacy for the hypothetical dog in the diets generated by AI chatbots, for beef- and chicken-based formulations^1^.

Model	Fat (g)	Linoleic acid n-6 (g)	Linolenic acid coverage (%)	Linolenic acid n-3 (g)	n-6:n-3 ratio	Status ratio^2^
Requirement	13.80	3.80	-	-	-	-
*Beef*						
Manus	42.90	1.50	38.5%	0.10	16.05	Elevated
ChatGPT 1	45.00	3.30	85.1%	0.70	4.88	Adequate
ChatGPT 2	44.00	1.90	49.4%	0.60	3.27	Adequate
DeepSeek	41.00	1.20	28.3%	0.00	0.00	Low
Gemini	41.00	2.40	64.7%	0.44	5.32	Adequate
*Chicken*						
Manus 1	26.90	2.90	76.50%	0.19	20.70	Elevated
Manus 2	49.75	8.02	210.00%	0.39	17.03	Elevated
ChatGPT 1	31.61	5.21	136.00%	9.34	0.56	Low
ChatGPT 2	32.98	3.06	80.20%	0.88	3.49	Adequate
DeepSeek	41.18	8.13	212.00%	0.00	16.25	Elevated
Gemini	27.04	2.47	64.70%	0.19	11.45	Elevated

1 considering the recommendations of FEDIAF (2024).

2 Suitable ratio: 2:1 to 8:1 (FEDIAF, 2024), <2 (low), ≥2;≤8 (adequate), >8 (elevated).

### 3.5 Essential amino acid profile

Compliance with the essential amino acid recommendations established by FEDIAF (2024) was generally adequate across the evaluated diets. Only two inadequacies were identified: a valine deficiency in the beef-based diet formulated by DeepSeek and a histidine deficiency in the chicken-thigh-based diet generated by Manus2 (Table 8). All other essential amino acids met or exceeded the minimum recommended levels in the remaining formulations.

**Table 8:** Amino acid requirements and levels of adequacy for the hypothetical dog in the diets generated by AI chatbots, for beef- and chicken-based formulations.

Model	Arg (g)	His (g)	Iso (g)	Leu (g)	Lys (g)	Met (g)	Met+Cys (g)	Phe (g)	Phe+Thy (g)	The (g)		Try (g)	Val (g)	AC¹ (%)
*Requirement*	1.51	0.67	1.33	2.37	1.22	1.16	2.21	1.56	2.58	1.51	0.49		1.71	100
*Beef*														
Manus	8.16	4.08	5.65	9.94	10.52	3.38	4.81	4.92	8.99	4.97	0.89		6.14	100
ChatGPT 1	6.53	3.18	4.55	7.99	8.29	2.58	3.89	4.01	7.22	4.05	0.68		5.04	100
ChatGPT 2	6.8	3.31	4.73	8.32	8.62	2.68	4.02	4.17	7.51	4.22	0.71		5.21	100
DeepSeek	6.2	3.44	4.98	8.77	9.07	2.79	4.33	4.52	8.1	4.55	0.83		**0.34**	91,7
Gemini	6.89	3.66	4.32	7.86	7.98	3.34	3.44	4.07	7.61	3.81	1.1		4.69	100
*Chicken*														
Manus 1	9.59	4.97	8.11	11.8	13.27	4.44	6.24	6.19	11.5	6.56	1.82		7.72	100
Manus 2	7.02	**0.56**	5.53	8.27	9.16	3.1	4.39	4.35	8.05	4.59	1.26		5.43	91,7
ChatGPT 1	8.04	4.11	6.98	9.97	11.2	3.64	5.35	5.35	9.8	5.66	1.58		6.67	100
ChatGPT 2	8.39	4.29	7.29	10.4	11.7	3.8	5.58	5.6	10.2	5.9	1.6		6.96	100
DeepSeek	6.63	2.9	5.03	7.9	8.6	2.65	3.8	4.16	7.8	4.42	1.18		4.5	100
Gemini	7.14	3.54	5.96	8.8	9.76	3.15	4.66	4.68	8.6	4.9	1.36		5.7	100

AC: actual compliance.

Arg: Arginine; His: Histidina; Iso: Isoleucine; Leu: Leucine; Lys: Lysine; Met: Methionine; Met+Cys: Methionine+cystine; Phe: Phenylalanine; Phe+Thy: Phenylalanine+tyrosine; Thr: Threonine; Try: Tryptophan; Val: Valine.

**Featured score:** Did not meet the minimum recommended score for the category.

1 AC (%): This is the percentage that represents compliance with all minimum requirements of the evaluated diets.

### 3.6 Mineral adequacy

Mineral adequacy varied markedly among the diets generated by the AI models when evaluated against the FEDIAF (2024) recommendations. Complete absence of chlorine was identified in all 11 diets (100%). The Ca:P ratio was outside the recommended range in 7 of 11 diets (63.6%). Copper, iodine, and magnesium were below recommendations in 7 of 11 diets (63.6%). Phosphorus and zinc were deficient in 8 of 11 diets (72.7%). Sodium and iron were deficient in 3 of 11 diets (27.3%). Potassium was deficient in 2 of 11 diets (18.2%). Selenium was deficient in 1 of 11 diets (9.1%). Manganese was deficient in 5 of 11 diets (45.5%). (Table 9).

**Table 9:** Mineral requirements and levels of adequacy for the hypothetical dog in the diets generated by AI chatbots, for beef- and chicken-based formulations.

Model	Ca (g)	P (g)	Ca:P (g)	K (g)	Na (g)	Cl (g)	Mg (g)	Cu (mg)	I (mg)	Fe (mg)	Mg (mg)	Se (mg)	Zn (mg)	AC^3^ (%)
*Requirement*	1.45^1^ 6.25^2^	1.16^1^ 4.0^2^	1^1^ 2^2^	1.45	0.29	0.43	0.2	2.08	0.3	10.4	1.67	0.087	20.8	100
*Beef*														
Manus	**0.05**	1.21	**0.04**	1.85	0.31	**0**	**0.13**	2.2	**0**	15.03	**0.21**	0.2	29.35	58.3
ChatGPT 1	**1.09**	**1.01**	1.08	1.79	**0.23**	**0**	**0.18**	**1.44**	0.58	10.53	2.48	0.34	24.97	50.0
ChatGPT 2	**1.13**	**1.05**	1.08	1.87	**0.24**	**0**	**0.19**	**1.5**	0.61	10.96	2.58	0.35	25.98	50.0
DeepSeek	4.03	1.5	**2.7**	3.31	0.47	**0**	0.2	6.2	**0**	16.74	**0.69**	0.17	**4.72**	58.3
Gemini	**0.08**	**0.81**	**0.1**	**1.15**	**0.2**	**0**	**0.17**	2.59	**0**	11.76	2.11	**0.03**	20.27	25.0
*Chicken*														
Manus 1	**0.1**	1.29	**0.08**	1.52	0.44	**0**	**0.16**	2.25	**0**	**10**	**0.26**	0.16	**7.48**	33.3
Manus 2	**0.07**	**0.77**	**0.09**	**1.02**	0.38	**0**	**0**	**2**	**0**	**9.62**	**0**	0.1	**12.66**	16.7
ChatGPT 1	**1.28**	**1.08**	1.19	1.88	0.4	**0**	0.2	**1.35**	0.64	**6.46**	1.76	0.32	**5.65**	41.7
ChatGPT 2	**1.33**	**1.13**	1.19	1.96	0.42	**0**	0.21	**1.41**	0.67	**6.74**	1.83	0.33	**5.9**	41.7
DeepSeek	**0.32**	**1.12**	**0.29**	2.58	0.52	**0**	0.26	**1.66**	**0**	22.67	2.28	0.13	20.37	66.7
Gemini	**0.19**	**0.98**	**0.19**	3.09	0.3	**0**	**0.16**	**0.64**	**0**	**6.21**	**1.31**	0.14	**5.7**	25.0

AC: actual compliance.

**Featured score:** Did not meet the minimum recommended score for the category or exceeded the maximum (when applicable).

1 Minimum recommended mineral content according to FEDIAF (2024);.

2 Maximum recommended mineral content according to FEDIAF (2024);.

3 AC (%): This is the percentage that represents compliance with all minimum requirements of the evaluated diets.

### 3.7 Vitamin adequacy

The observed deficiencies were vitamin A and B12 in 1 of the 11 diets (9.1%), vitamin D deficiency in 7 of the 11 diets (63.7%), vitamin E deficiency in 6 of the 11 diets (54.5%), vitamin B1 and B2 deficiency in 2 of the 11 diets (18.2%), vitamin B8 deficiency in 9 of the 11 diets (81.8%), and vitamin B9 deficieny in 5 of the 11 diets (45.5%) (Table 10).

**Table 10:** Vitamin requirements and levels of adequacy for the hypothetical dog in the diets generated by AI chatbots, for beef- and chicken-based formulations.

Model	Vit.A (UI)	Vit.D (UI)	Vit.E (UI)	Vit.B1 (mg)	Vit.B2 (mg)	Vit.B3 (mg)	Vit.B5 (mg)	Vit.B6 (mg)	Vit.B8 (mg)	Vit.B9 (mg)	Vit.B12 (mg)	AC^3^ (%)
*Requirement*	1,754^1^ 100,000^2^	159^1^ 800^2^	10.4	0.62	1.74	4.74	4.11	0.42	474	0.0747	0.00968	100
*Beef*												
Manus	24,119	**4.14**	**2.14**	1.41	2.52	33.27	6.38	2.53	499.9	**0.03**	0.04	72.7
ChatGPT 1	20,306	172.4	13.57	2.39	2.65	22.17	7.92	3.55	**410.59**	0.26	0.01	90.9
ChatGPT 2	21,131	179.4	14.12	2.49	2.76	23.07	8.24	3.7	**427.27**	0.27	0.01	90.9
DeepSeek	49,883	**27.08**	**10.11**	0.82	3.25	35.91	9.85	3.69	630.24	0.36	0.06	81.8
Gemini	32,884	**25.22**	35.26	**0.46**	**1.55**	16.46	5.08	1.04	**41.75**	**0.07**	0.04	54.5
*Chicken*												
Manus 1	26,706	**4.57**	**2.83**	1.63	2.34	73.72	8.21	3.17	467.85	**0.03**	0.04	63.6
Manus 2	22,988	**22.96**	**2.41**	1.31	2.28	26.52	6.59	1.14	**298.74**	**0.03**	0.03	63.6
ChatGPT 1	31,114	189.76	13.82	2.65	2.6	58.38	10.84	4.52	**433.31**	0.39	0.01	90.9
ChatGPT 2	32,461	197.97	14.42	2.77	2.71	60.9	11.31	4.72	**452.06**	0.4	0.01	90.9
DeepSeek	**57.04**	**0**	**8.73**	0.85	2.78	22.34	11.54	2.81	**146.53**	0.61	0.02	72.7
Gemini	45,972	**0**	**3,18**	**0,5**	**0,65**	49.28	4.4	2.65	**356.58**	**0.05**	**0**	36.4

AC: actual compliance.

**Featured score:** Did not meet the minimum recommended score for the category or exceeded the maximum (when applicable).

1 Minimum recommended vitamin intake according to FEDIAF (2024).

2 Maximum recommended vitamin intake according to FEDIAF (2024).

3 AC (%): This is the percentage that represents compliance with all minimum requirements of the evaluated diets.

### 3.8 Consolidated analysis of nutritional adequacy

Overall, 61.3% of the analyzed nutrients were below the minimum levels recommended by FEDIAF (2024), whereas only 38.7% met adequacy. The general compliance across all nutrients, grouped into seven categories (energy, protein, amino acids, ether extract, linoleic acid, minerals, and vitamins) and stratified by AI model, is summarized (Table 11).

**Table 11:** Consolidated compliance assessment of FEDIAF (2024) nutritional requirements for the hypothetical dog by diets generated by artificial intelligence models.

Model	ME	Crude protein	Amino acid	EE	Linolenic acid	Minerals	Vitamins	Average^1^
*Beef*								
Manus	76.3	100	100	100	38.5	58.3	72.7	78
ChatGPT 1	76.6	100	100	100	85.1	50.0	90.9	86.1
ChatGPT 2	73.6	100	100	100	49.4	50.0	90.9	80.6
DeepSeek	100	100	91.7	100	28.3	58.3	81.8	80
Gemini	93.0	100	100	100	64.7	25.0	54.5	76.7
*Chicken*								
Manus 1	69.1	100	100	100	76.5	33.3	63.6	77.5
Manus 2	81.2	100	91.7	100	100	16.7	63.6	79
ChatGPT 1	69.5	100	100	100	100	41.7	90.9	86
ChatGPT 2	66.7	100	100	100	80.2	41.7	90.9	82.8
DeepSeek	100	100	100	100	100	66.7	72.7	91.3
Gemini	85.4	100	100	100	64.7	25.0	36.4	73
Average^1^	81	100	98.5	100	72.7	42.4	73.5	-
AC^2^	18	100	81	100	27.3	0	0	-

AC: actual compliance; ME: metabolizable energy EE: ethereal extract.

1 Average (row): consolidated mean compliance percentage across all nutritional categories for each AI model. Average (column): mean compliance percentage across all AI models for each nutritional category.

2 Represents the percentage of diets that fulfilled all minimum recommended requirements.

In general, adequate compliance was observed for crude protein and ether extract in all formulations, while ME requirement was not met in most models. The greatest deficits were identified for linoleic acid, minerals, and vitamins with average compliance percentages of 72.7%, 42.4%, and 73.5%, respectively. Among the models, DeepSeek showed the highest average adequacy percentages, especially in the chicken-based diet, while other models demonstrated significant inconsistencies.

## 4 Discussion

### 4.1 Energy inconsistency and metabolic risk

It is important to emphasize that this was a theoretical, in silico study, and no dogs were subjected to these diets.

The results of this study revealed a systematic failure of AI models to adequately calibrate prescribed food volume and dietary energy density, resulting in under- or overestimation of energy supply with potential metabolic implications. Energy insufficiency, observed in 81% of the formulated diets, could lead to energy undernutrition if these diets were chronically fed to an animal with the characteristics specified in the prompt (Case et al., 2011). Inadequate energy intake may promote the mobilization of body reserves, progressive loss of body condition, and reduction of muscle mass, as reported in experimental studies involving dogs fed diets with lower energy density (Sun et al., 2023).

Overfeeding, which occurred with the chicken-based diet formulated by DeepSeek, would lead to severe overweight and obesity (Case et al., 2011). Canine obesity is recognized as the most prevalent nutritional disorder in developed countries, affecting 25–45% of the canine population, and is associated with multiple comorbidities (German, 2006), in addition to reduced life expectancy (Kealy et al., 2002).

### 4.2 Lipid imbalances and inflammation

The results revealed three recurring patterns in the lipid composition of the evaluated diets: i) marked deficiency of linoleic acid (omega-6), ii) omega-6:omega-3 ratios outside the recommended ranges, and iii) high variability in the selection of lipid sources used in the formulations. These findings indicate inadequacies in meeting nutritional recommendations for essential fatty acids. Such imbalances in fatty acid composition can result in alterations in skin barrier integrity, immune function, and inflammatory response mechanisms, with potential negative impacts on the health and quality of life of the animals, as described in the literature (Bauer, 2006; Lenox and Bauer, 2013).

#### 4.2.1 Linoleic acid (LA) deficiency

Linoleic acid (LA; 18:2 n-6) is the only fatty acid considered strictly essential for dogs, since they lack the Δ12-desaturase enzyme necessary for its endogenous synthesis (Bauer, 2006; NRC, 2006). The results of this study demonstrated widespread undersupply of linoleic acid in 72% of the diets formulated by AI models. Clinical linoleic acid deficiency initially manifests as a dull, dry, and brittle coat, progressing to focal alopecia (especially in areas of friction), erythema, pruritus, and, in severe cases, generalized exfoliative dermatitis (Campbell, 1993).

#### 4.2.2 Omega-6 to omega-3 ratio

The omega-6:omega-3 (n-6:n-3) ratio is a critical determinant of the dietary inflammatory profile, influencing the production of lipid mediators with opposing biological activities (Bauer, 2006). The ideal range established for dogs is between 2:1 and 8:1 (FEDIAF, 2024) based on studies demonstrating that ratios within this range optimize the synthesis of anti-inflammatory eicosanoids without compromising the availability of precursors for physiological processes that require pro-inflammatory mediators (Kar et al., 2024). The results of this study identified high ratios in multiple diets: the Manus1 and Manus2 (chicken) formulations with 20.70:1 and 17.03:1, DeepSeek (chicken) with 16.25:1, and Gemini (chicken) with 11.45:1. These ratios exceed the recommended upper limit, characterizing a biochemically pro-inflammatory environment.

Interestingly, some diets that presented adequate n-6:n-3 ratios (within the range of 2:1 to 8:1) still showed absolute linoleic acid deficiency. This paradox is exemplified by the ChatGPT1 beef-based diet, which presented a ratio of 4.87:1 (ideal), but a deficit of −15% in linoleic acid. From a clinical point of view, an ideal n-6:n-3 ratio does not compensate for absolute linoleic acid deficiency. The animal may still develop fatty acid-responsive dermatosis, as the total amount of LA is insufficient to maintain basal metabolic needs (Campbell, 1993).

#### 4.2.3 Choice of lipid source

The selection of lipid sources in dietary formulations should consider three simultaneous criteria: i) adequate supply of linoleic acid, ii) contribution of EPA+DHA when applicable, and iii) oxidative stability.

The models used only single lipid sources, namely olive oil, flaxseed oil, soybean oil, coconut oil, or isolated fish oil; however, the ideal strategy for homemade diets involves combining sources, such as soybean or canola oil as a base to meet linoleic acid requirements, supplemented with fish oil (1–2% of the diet) to optimize the n-6:n-3 ratio if necessary and direct supply of EPA+DHA (Bauer, 2011; Lenox and Bauer, 2013).

The failure of chatbots to balance fatty acids is not exclusive to veterinary nutrition. Kaçar et al. (2025), when evaluating human diets generated by ChatGPT, Microsoft Copilot and Gemini, identified average n-6:n-3 ratios significantly above the recommendation for humans. Papastratis et al. (2024) reported deficits of 20% in fatty acids in omnivorous human diets formulated by AI.

#### 4.2.4 The ChatGPT case

This inconsistency reflects a critical limitation of the model in understanding the biochemical complementarity of lipid sources. By offering single oil sources as equivalent alternatives, it demonstrates an inability to differentiate specific nutritional functions: olive oil, although a source of beneficial monounsaturated fatty acids, contains only 8–12% linoleic acid versus 50–60% in soybean oil (Bauer, 2006); flaxseed oil, despite being rich in alpha-linolenic acid (ALA), has limited conversion to EPA and DHA in dogs (5–15% and <1%, respectively), making it unsuitable as a sole source of omega-3 (Bauer, 2006). This flaw exemplifies how linguistic models generate recommendations that appear variable but result in predictable nutritional imbalances for trained professionals. This reinforces the thesis that linguistic sophistication does not translate into structured nutritional reasoning.

### 4.3 Mineral deficiencies

Mineral analysis revealed the most severe and systematic deficiencies among all AI-formulated diets with all models failing to meet the minimum recommended levels for multiple essential minerals. This finding is particularly alarming, since mineral metabolism in dogs is highly sensitive to imbalances, and the clinical consequences of prolonged deficiencies are well documented in the veterinary literature (Tryfonidou et al., 2003; Zafalon et al., 2020).

#### 4.3.1 Calcium, phosphorus, and their ratio

The calcium:phosphorus (Ca:P) ratio is the most critical mineral parameter identified in this study. FEDIAF (2024) and NRC (2006) establish that the ideal Ca:P ratio should be between 1:1 and 2:1 for adequate maintenance of mineral homeostasis. Ratios lower than 0.5:1, as observed in 40% of the diets analyzed, are directly associated with the development of secondary nutritional hyperparathyroidism (Krook and Whalen, 2010). Clinical studies demonstrate that dogs fed diets unbalanced in Ca:P for periods longer than 4–6 weeks begin to show clinical signs of secondary nutritional hyperparathyroidism, including lameness, reluctance to move, bone pain, and, in puppies, growth retardation and angular limb deformities (Schoenmakers et al., 2000).

Pedrinelli et al. (2017), when analyzing 200 homemade dog food recipes published in books and websites, identified that 95% presented inadequacies in the Ca:P ratio, demonstrating that this is a recurring error even in formulations prepared by laypeople. The work of Pedrinelli et al. (2017) is particularly relevant to understanding the shortcomings of the AI models evaluated in this study. LLMs are trained on vast datasets of text extracted from the internet, including blogs, forums, non-specialized websites, and recipes published by pet owners without technical training (Devlin et al., 2019). If 95% of the recipes available online present a Ca:P imbalance, the AI models statistically learn that diets with inadequate ratios are the dominant pattern in their training data, not the exception (Ji et al., 2023). Consequently, when generating new formulations, chatbots replicate the systematic errors present in the textual corpus, then perpetuate nutritional inadequacies under the guise of technical authority (Onay et al., 2025). This phenomenon explains why three of the four models evaluated (Manus, DeepSeek, and Gemini) critically failed in mineral balancing: not due to a limitation of theoretical "knowledge" about the importance of the Ca:P ratio, but due to the absence of mechanisms that translate abstract nutritional principles into precise quantitative calculations during formulation (Kumar and Sharma, 2025).

In addition to the relational imbalance, these diets presented absolute calcium deficiencies. In young dogs, severe calcium deficiency during the growth phase can result in rickets, characterized by failure in the mineralization of the organic bone matrix (osteoid), leading to deformities such as limb bowing, epiphyseal widening, and spontaneous fractures (Krook and Whalen, 2010). In adults, the analogous condition is osteomalacia, which, although less dramatic in its clinical presentation, results in chronic bone pain, muscle weakness, and an increased risk of fragility fractures (Schoenmakers et al., 2000).

Phosphorus levels were relatively closer to recommendations. Phosphorus is naturally abundant in animal protein sources, especially in muscle and visceral tissues (NRC, 2006), which explains its greater presence in the formulated diets, since all models met the minimum protein requirement. Relative excess phosphorus not only reduces intestinal calcium absorption by forming insoluble complexes (calcium phosphate) in the intestinal lumen, but also directly stimulates PTH secretion even in normocalcemia states (Tryfonidou et al., 2003). Additionally, high serum phosphorus levels suppress renal synthesis of calcitriol (the active form of vitamin D), further compromising intestinal calcium absorption and creating a vicious cycle of hypocalcemia and hyperparathyroidism (DiBartola, 2022).

#### 4.3.2 Chlorine

The complete absence of chlorine (0% fulfillment) in all formulated diets represents an even more basic failure to understand. Chlorine, supplied primarily through sodium chloride (table salt), is the main extracellular anion and plays vital roles in acid-base balance, nerve transmission, and hydrochloric acid secretion in the stomach. Chlorine deficiency results in hypochloremic metabolic alkalosis, characterized by elevated blood pH, compensatory hypokalemia, and impaired renal function (DiBartola, 2022).

#### 4.3.3 Other minerals

Zinc deficiency in dogs results in a dermatological syndrome characterized by hyperkeratosis, alopecia, crusted lesions, and increased susceptibility to secondary infections (NRC, 2006). Copper is essential for hemoglobin synthesis, collagen formation, and central nervous system function, and its deficiency results in microcytic anemia, bone fragility, and coat depigmentation (Diaz et al., 2015).

Potassium deficiency causes generalized muscle weakness, lethargy, and anorexia (DiBartola, 2022). Severe hypokalemia can result in potentially fatal cardiac arrhythmias (Ettinger et al., 2017). Magnesium deficiency is associated with muscle tremors, tetany, and seizures (Fascetti and Delaney, 2012). Chronic deficiency can result in anorexia, growth retardation, and soft tissue calcification (NRC, 2006).

Iodine deficiency results in hypothyroidism with reduced basal metabolism and weight gain (Mooney, 2011). Goiter, a compensatory enlargement of the thyroid gland, is a classic manifestation of chronic deficiency (Johnson, 2005).

#### 4.3.4 Supplementation

The analysis of the diets formulated by the four AI models showed an absence or inadequacy of specific vitamin-mineral supplementation for homemade diets. Supplementation is not an optional component in natural diets, but rather an absolute necessity to fill the nutritional gaps inherent in whole foods (Saad and França, 2010). The Manus, DeepSeek, and Gemini models did not include any form of vitamin-mineral supplementation, depending exclusively on the nutrients naturally present in the ingredients, an approach that the literature demonstrates to be invariably insufficient (Pedrinelli et al., 2022; Heinze et al., 2012).

ChatGpt, however, included eggshell (1.2 *g*/day), which represents a partial recognition of the need for supplemental calcium. Eggshell is composed of approximately 40% elemental calcium (as calcium carbonate), providing about 480 mg of calcium per gram of ground shell (NRC, 2006). The supplement Vetnil® was recommended, a Brazilian company that manufactures multiple veterinary products, including various vitamin and mineral supplements; however, the recommended supplement is not intended for balancing homemade diets, but rather as support for hospitalized dogs.

Previous studies consistently demonstrate inadequacies in unsupplemented homemade diets. Stockman et al. (2013), when evaluating 67 homemade diets prepared by pet owners, identified that unsupplemented diets were deficient in at least one nutrient.. Pedrinelli et al. (2017), in an analysis of 200 published recipes, reported that 95% presented at least one critical nutritional inadequacy with predominant deficits in calcium (94%), vitamin D (78%) and zinc (65%). This finding reinforces that supplementation is not an excessive precaution, but rather an essential intervention for the nutritional safety of homemade diets.

## 5 Raw diets

The suggestion of raw ingredients by the DeepSeek model, particularly raw beef (round steak and offal) and raw chicken components (ground bone-in thigh/drumstick), introduces a risk that transcends the nutritional inadequacies previously discussed. Although raw diets (raw feeding) have gained popularity among pet owners in recent years, based on arguments of naturalness and ancestry, the scientific community maintains significant reservations regarding their microbiological safety (Bernaquez et al., 2025).

Raw meats, even when acquired for human consumption, present a significant microbial load, including zoonotic pathogens such as *Salmonella* spp., *Escherichia coli* O157:H7, *Listeria monocytogenes, Campylobacter* spp., and *Clostridium perfringens* (Weese et al., 2005). Prevalent studies show that 20% of commercially available raw meat samples are contaminated with Salmonella, with variations depending on the animal species, cut, and processing conditions (Van Bree et al., 2018).

The concern is not limited to the direct risk to the dog, which has some resistance to enteric pathogens due to its high gastric acidity (pH∼1–2), but extends to the risk of zoonotic transmission to living humans. Dogs fed raw diets excrete viable pathogens in their feces for extended periods, contaminate the domestic environment through saliva and fur, and represent a source of infection for children, the elderly, and immunocompromised individuals (Schlesinger and Joffe, 2011). Bernaquez et al. (2025) identified 14 documented outbreaks of salmonellosis in humans associated with contact with dogs fed raw diets, reinforcing the public health relevance of this practice.

In addition to bacteria, raw meat can contain nematode larvae (*Toxocara* spp.), tapeworm metacestodes (*Taenia* spp., *Echinococcus* spp.) and protozoan cysts (*Toxoplasma gondii, Neospora caninum*) (Wayszceyk and Goulart, 2021). Commercial freezing (−18 °C for 24–72 hours) reduces but does not completely eliminate some of these agents. The absence of adequate heat treatment (cooking at ≥75 °C for at least 15 min) maintains the viability of infective forms, exposing the animal to parasitic infections that may have severe clinical manifestations or remain subclinical, but with zoonotic potential (Van Bree et al., 2018).

The inclusion of ground bone thigh/drumstick by the DeepSeek model deserves additional attention. Although raw bones are less likely to splinter than cooked bones, they still pose a risk of esophageal and gastroenteric obstruction and intestinal perforation, especially in dogs that consume food quickly without proper chewing (Freeman et al., 2013).

## 6 Limitations and potential for professional use assisted by AI

### 6.1 Simplified prompt design and real-world lay use

This study intentionally employed a simple, non-technical prompt (“Create two complete and balanced diets…”), representative of the type of request commonly made by lay pet owners interacting with AI chatbots without prior veterinary consultation (OVC Pet Nutrition, 2024). No detailed specifications regarding metabolizable energy, amino acid profile, mineral ratios, or supplementation strategies were provided.

This methodological choice reflects the most prevalent real-world use scenario and therefore prioritizes real-world applicability over optimized prompt engineering. However, it is plausible that highly detailed prompts developed by trained professionals could generate nutritionally superior outputs. For example, a veterinary nutritionist could specify caloric targets, Ca:P ratios, essential fatty acid requirements, mandatory vitamin-mineral supplementation, and target energy density. Under such conditions, AI-generated outputs could serve as preliminary drafts to be reviewed, corrected, and validated using specialized formulation software (Kumar and Sharma, 2025).

Importantly, this study evaluated single-prompt interactions, which likely reflect typical lay use. Iterative prompting, in which users progressively refine outputs after identifying deficiencies, could theoretically improve formulations. For example, a user could request inclusion of a vitamin-mineral supplement after receiving an unsupplemented recipe. However, this process presupposes that the user is already capable of recognizing nutritional inadequacies, a level of technical knowledge that cannot reasonably be assumed in the general population of pet owners.

### 6.2 Limitations of nutritional analysis

Another limitation is that nutritional composition was estimated using SuperCracPet® software based on USDA food composition databases. These calculations represent standardized estimated nutrient values rather than direct laboratory analysis of prepared diets. Actual nutrient composition may vary according to ingredient origin, processing, cooking temperature, preparation method, storage conditions, and batch-to-batch variation.

Therefore, laboratory analysis of physically prepared diets would be necessary to confirm the true nutrient composition of the recipes generated by the AI models.

### 6.3 Temporal limitation related to rapid AI evolution

A limitation inherent to studies evaluating generative AI systems is the rapid pace of model evolution. The four models analyzed in this study were evaluated using their publicly available versions in December 2025 (Manus, ChatGPT-4o, Gemini 2.0 Flash Experimental, and DeepSeek-V3). Subsequent updates in architecture, parameter refinement, reasoning capacity, or integration with external nutritional databases may substantially alter model performance in dietary formulation tasks.

Accordingly, the present findings represent a time-specific evaluation and may not reflect the capabilities of future model versions. Nevertheless, this limitation does not invalidate the current results, which document the actual performance observed under real-world conditions at the time of analysis. Instead, it highlights the need for continuous re-evaluation as AI systems evolve.

### 6.4 Clinical risks in diseased animals

This study evaluated diet formulation for a healthy adult dog. However, the risks associated with AI-generated diets are likely to be substantially greater in animals with underlying medical conditions. Diseases such as chronic kidney disease, hepatic encephalopathy, pancreatitis, urolithiasis, food allergies, and diabetes mellitus require highly individualized nutritional management with precise control of specific nutrients based on clinical and laboratory assessment.

Given that the AI systems evaluated in this study did not consistently achieve basic nutritional adequacy even for a healthy animal, their unsupervised use in therapeutic diet formulation raises significant clinical concerns. Inadequate nutritional management in diseased animals could contribute to disease progression, metabolic instability, or severe nutrient imbalances.

### 6.5 Professional and ethical implications

A broader concern associated with AI-generated dietary tools is the potential displacement of veterinary consultation. Because AI systems frequently generate well-structured and apparently authoritative dietary plans, pet owners may perceive these outputs as equivalent or superior to professional veterinary guidance, particularly due to their immediate availability and absence of direct financial cost.

This dynamic may undermine the veterinarian-client-patient relationship and create additional burdens for veterinary teams, which may subsequently need to identify and correct nutritional errors generated by AI systems. Similar concerns have been described in human medicine, where AI-assisted self-diagnosis and self-treatment are associated with increased risk of clinical error when professional oversight is absent (Thirunavukarasu et al., 2023).

Therefore, public communication regarding the limitations of AI-generated nutritional advice, together with reinforcement of the indispensable role of qualified veterinary professionals, should be prioritized by professional organizations and regulatory agencies.

### 6.6 Future perspectives for AI-assisted veterinary nutrition

Future advances in quantitative reasoning, integration with validated nutritional databases, and hybrid systems combining large language models with dedicated nutritional calculation engines may substantially improve formulation quality.

However, even if future systems achieve greater technical accuracy, professional supervision will likely remain indispensable for at least two reasons. First, appropriate dietary formulation requires individualized clinical assessment, including body condition, medical history, lifestyle, owner compliance, and therapeutic objectives, factors that cannot be fully captured through text prompts alone. Second, ethical and legal responsibility for dietary prescription in animals must remain under the authority of licensed professionals.

Accordingly, the most appropriate future role of AI in veterinary nutrition is likely to be that of a professional support tool rather than an autonomous substitute for veterinary expertise.

## 7 Conclusions

The evaluated language models (Manus, ChatGPT, DeepSeek, and Gemini) demonstrated systematic inadequacy in the formulation of nutritionally complete diets for dogs, with 61.3% of the nutrients below the minimum levels recommended by FEDIAF. This resulted in prescriptions with severe nutritional deficiencies, potentially compromising bone, skin, immune, and metabolic health. In this context, artificial intelligence may represent a valuable tool for advancing canine diet formulation; however, its safe and effective application depends on professional oversight. Technical expertise in canine nutrition remains essential to critically evaluate AI-generated recommendations, adjust formulations, and ensure balanced diets that safeguard animal health and welfare.

## Ethical statement

This study did not involve live animals, biological samples, or human participants. The analysis was exclusively based on textual outputs generated by publicly accessible artificial intelligence systems. Therefore, according to institutional and national regulations, ethical approval was not required.

## Declaration of generative AI and AI-assisted technologies in the manuscript preparation process

During the preparation of this work, the authors used the AIs Manus, DeepSeek, Gemini, and ChatGpt as the central object of study, not as a methodological aid or writing tool. All nutritional analysis, critical interpretation, scientific discussion, and writing of the article were carried out exclusively by the authors. The authors assume full responsibility for the accuracy, integrity, and interpretation of all data presented in this manuscript.

## Funding sources

This work was funded by the Coordenação de Aperfeiçoamento de Pessoal de Nível Superior (CAPES) of Brazil, under grant number 88887.143769/2025–00. The authors also acknowledge CAPES for supporting the open-access publication of this article through the CAPES Transformative Agreement.

## CRediT authorship contribution statement

**M.B.S. Rosa:** Writing – original draft, Software, Project administration, Methodology, Investigation, Data curation, Conceptualization. **J. França:** Writing – review & editing. **M.T.S. Siqueira:** Writing – review & editing. **C. Raineri:** Writing – review & editing, Supervision, Methodology, Investigation.

## Declaration of competing interest

The authors declare that they have no known competing financial interests or personal relationships that could have appeared to influence the work reported in this paper.

## Glossary

AAFCO: Association of American Feed Control Officials
AI: Artificial Intelligence
BW: body weight
DER: daily energy requirement
FEDIAF: European Pet Food Industry Federation
LLM: Large Language Models
ME: metabolizable energy
ML: machine learning
NRC: National Research Council
USDA: United States Department of Agriculture
